# Prevalence of and Risk Factors for Lead Poisoning in Young Children in Bangladesh

**DOI:** 10.3329/jhpn.v30i4.13292

**Published:** 2012-12

**Authors:** Amal K. Mitra, Emmanuel Ahua, Pradip K. Saha

**Affiliations:** ^1^Department of Community Health Sciences, University of Southern Mississippi, Hattiesburg, Mississippi, USA;; ^2^People's University of Bangladesh, Lalmatia, Dhaka, Bangladesh

**Keywords:** Child, Cross-sectional studies, Lead poisoning, Risk factors, Bangladesh

## Abstract

Lead poisoning is a major public-health problem in Bangladesh. A cross-sectional study was conducted to determine the extent of and risk factors for elevated blood lead levels (BLLs) in children in Bangladesh during September 2007–July 2009. The study included 919 children aged less than 16 years. The children were recruited from six urban locations in Dhaka and one rural area in Chirirbandar, Dinajpur. In total, 495 (54%) children had high BLLs (>10 µg/dL), with higher BLLs observed among children aged 5-9 years compared to children of other ages (p<0.001). The BLLs among children in urban Dhaka were significantly higher than those in rural areas (13.45±8.21 µg/dL vs 7.29±6.25 µg/dL, p<0.001). The high BLLs correlated with low body mass index (r=-0.23, p<0.001) and low haemoglobin status (r=-0.10, p=0.02). On bivariate analysis, proximity to industry (p<0.001), drinking-water from municipal supply or tubewell (p<0.001), brass or lead water-taps (p<0.001), use of melamine plate (p=0.001), and indigenous medicinal (*kabiraji*) treatments (p=0.004) significantly correlated with higher BLLs. Proximity to industry and the use of indigenous medicines remained significant predictors of high BLLs after controlling for the confounders. Several risk factors appropriate for future educational interventions to prevent exposure to lead poisoning were identified.

## INTRODUCTION

Lead is the number one environmental threat to the health of children (1). Some populations and geographic areas still remain at a disproportionately-high risk of exposure to lead (2). In children, the blood lead levels (BLLs) as low as 10 μg/dL are associated with developmental delays, deficits in behavioural functioning, decreased stature, and diminished hearing acuity (3-5). The high BLLs (i.e. ≥70 µg/dL) can cause serious health effects, including seizures, coma, and death (6).

In 1991, the Centers for Disease Control and Prevention (CDC) recommended universal screening of all children aged 6-72 months because exposure to lead is still common in certain communities (7). Healthy People 2020, the nation's strategy for improving the health and well-being of all citizens, has established a goal of reducing the mean BLLs in children aged 1-5 year(s) by 10% from its existing levels (8).

Although Bangladesh adopted a policy to ban the sale of leaded gasoline in 1999, the risk of lead poisoning in young children is uncertain because of the absence of a lead-screening programme in the country. Specially, data on the BLLs are almost non-existent in rural Bangladesh. Results of a study among urban school children in Bangladesh showed high BLLs in approximately 90% of children (9). In another study, nearly all children tested in an industrial area at Tongi, Gazipur, had high BLLs (10). The major types of industries in the area, located 18 km northeast of Dhaka, included: battery, ceramic, pharmaceutical, cotton, jute, and plastic. Two major sources of lead poisoning identified in this area were industrial discharges and the use of leaded gasoline in the autorickshaw with two-stroke engines. These data are an indication of the alarming rates of lead poisoning in the country and provide evidence for the need for further studies to determine the extent of the problem in other urban and rural areas of Bangladesh.

The present study aimed to: (a) determine the prevalence of lead poisoning in preschool and school-age children in urban and rural communities of Bangladesh and (b) assess the risk factors associated with high BLLs in this population.

## MATERIALS AND METHODS

### Study subjects

The study was part of a Fulbright Scholar Research Program in Bangladesh conducted in two phases. The first part of the study was conducted during September 2007–July 2008, and the second part was conducted during May-July 2009. The study, which used a cross-sectional design, included children, aged less than 16 years, who were recruited from six schools and communities in urban Dhaka and one rural school in the northern area of Chiri-rbandar, Dinajpur.

### Collection of data

A team consisting of two epidemiologists, three laboratory technicians, and volunteers from the community collected data using a pretested questionnaire. The independent variables included: age, gender, income, height, weight, haemoglobin (Hb), iron, and blood group. The possible sources of lead investigated were: type of residence, proximity to highways and industries, use of pesticides, water sources and materials used for water faucets, type of serving-plate used, and indigenous treatments. Body-weights of children were measured using a bathroom scale, with an accuracy of 1 g while their heights were measured using a standard mechanical stadiometer, with an accuracy of 1 cm. Body mass index (BMI) was computed as weight (kg), divided by the square of height in metre.

### Measurements of blood lead

Samples of venous blood (0.5 mL) were collected by aseptic measures, transported using heparin tubes, and processed within 24 hours after collection. The BLLs were measured using a portable LeadCare II Blood Lead Test instrument (ESA Inc., Chelmsford, MA, USA). LeadCare II is a CLIA (Clinical Laboratory Improvement Amendments)-waived test as it meets the requirements of precision and accuracy for lead in blood test analysis. This instrument, approved by the US Food and Drug Administration, is extensively used in health centres in the United States and in other countries (10,11). The device allows quick and accurate screening and test results in a few minutes and is well-suited for the community outreach settings. The reliability and the accuracy of the instrument have been reported earlier (11).

### Quality assurance and quality control

Fresh, unrefrigerated whole blood (without any clots) was used for analysis. All the test-kits were stored in a cool, dry place at 15-27 ºC, away from direct sunlight. Sensors were kept sealed in their containers until the sample was prepared and made ready to perform the test. The treatment reagent was used immediately after opening the tube. Expiration dates were checked for sensors, blood lead controls, and treatment reagents before use. Two levels of controls—low (Level 1) and high (Level 2)—were used at the beginning of each day the analyzer was used for testing blood, and all the control values were recorded on a quality-control log. The reportable range of the test was 3.3-65 µg/dL. The controls had the same lot number as the sensors, and the target values were provided right on the control vials.

The validity of data was ensured by taking the following measures: (a) to ensure the appropriate laboratory procedures in place, samples of four volunteers were analyzed at the field level; (b) all the blood samples were measured by a single person with specific training (AM); (c) the instrument was calibrated each time before running a sample for analysis; (d) LeadCare blood lead controls were analyzed on each occasion a new lot of test-kit was used; and (e) if the measurement of BLL was 30 µg/dL or more, the instrument was validated by calibration, and the test was repeated by analyzing a second blood sample.

### Statistical methods

Data were entered and cleaned using the SPSS for Windows software (version 18) (SPSS Inc., Chicago, IL, USA). The BLLs were classified into the following categories: <10 µg/dL; 10-14 µg/dL; 15-19 µg/dL; 20-44 µg/dL; and 45-69 µg/dL. The categories of the BLLs were compared among groups of urban and rural children by the chi-square test of independence. The mean BLLs were compared among the age-groups using one-way analysis of variance (ANOVA) with the Tukey's HSD test and 95% confidence intervals (CIs). The Student's ***t***-test or the Mann-Whitney test was used, depending on the distribution of data, for comparing the continuous variables between the two groups. Pearson's correlation was used for assessing correlation between the BLLs and other independent variables. A multiple regression test was performed to determine the predictors of BLLs after controlling for the confounders. A probability level of 0.05 or less was considered significant.

### Ethical aspects

The Institutional Review Board of the University of Southern Mississippi and the Bangladesh Medical Research Council approved the study. Informed consent was obtained from parents of younger children, and assent was obtained from older children (13-18 years) before enrollment. Approval was also obtained from the school authorities to provide health education to children on the prevention of lead exposure.

## RESULTS

In total, 921 children were enrolled, of whom two were dropped because of insufficient blood samples. Distribution of the remaining 919 samples was as follows: urban Dhaka (n=722) comprising Tongi industrial area (n=105), Jigatola Tannery (n=170), Mohakhali (n=244), Lalmatia (n=80), Mohammadpur (n=64), Uttara (n=59) and a rural area of Chirirbandar (n=197) (Fig.).

### Urban-rural difference in prevalence of elevated BLLs

Table 1 shows that 46% of the study subjects had normal BLLs. The proportion of children with high BLLs was significantly high in urban areas compared to rural areas (64% vs 15%). The BLL of 20 µg/dL or higher equalled 16.4% in urban children compared to 2.5% in rural children (p<0.001). The prevalence of elevated BLLs among the urban children was the highest (99%) in the Tongi industrial area, followed by Jigatola Tannery (91%), Mohakhali (58%), Lalmatia (50%), Mohammadpur (33%), and Uttara (5%) (Fig.). The mean±standard deviation (SD) of BLLs was significantly higher among the urban children than among the rural children [13.45±8.21 µg/dL vs 7.29±6.25 µg/dL, p<0.001, 95% confidence interval (CI) 5.10-7.22 µg/dL].

### Risk factors associated with elevated BLLs

The mean±SD of BLLs was the highest in children aged 5-9 years whereas this value was the lowest in children aged 10 years and above (13.58±8.67 vs 10.84±7.26 µg/dL, p<0.001) (Table 2). Results of Pearson's correlation analysis showed that the BLLs inversely and significantly correlated with an increasing age (r=-0.14, p<0.001) and lower BMI (r=-0.23, p<0.001) (Table 3). No significant association was observed between the BLLs and serum iron (r=-0.07, p=0.10) or Hb (r=-0.01, p=0.82). Children with blood group B–(n=7) had the highest BLLs (18.0±37.08 µg/dL), and those with blood group AB–(n=3) had the lowest BLLs (4.93±0.78 µg/dL, p=0.01). Several risk factors significantly correlated with higher BLLs (Table 4). These included: presence of industries in proximity to the residence (p<0.001); the use of tubewell water (p<0.001) or municipal water supply (p=0.02) compared to the use of home filters; water faucets made of brass, lead, or copper (p<0.001); the use of melamine plates (p<0.001); and the use of *kabiraji* (indigenous) medicines (p=0.004). When the type of residence (urban or rural) was controlled, the BLLs did not correlate significantly with municipal water supply (p=0.37). To further control for the confounders, a multiple regression analysis was performed to predict the BLLs. The proximity to industries (p=0.002) and the use of indigenous medicines (p=0.011) were the significant predictors of BLLs after controlling for the confounders.

**Fig.**
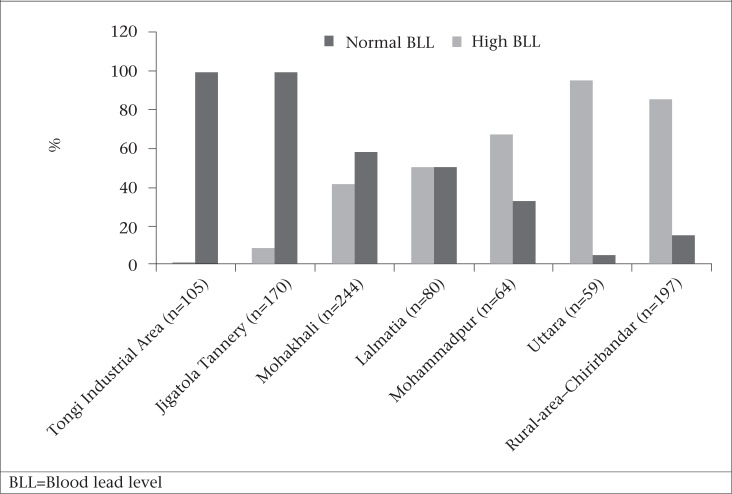
Distribution (%) of blood lead levels by location

**Table 1.** Blood lead levels in urban and rural children in Bangladesh

Lead level	Urban (n=722)	Rural (n=197)	Total (n=919)
<10 µg/dL	257 (35.6)	167 (84.8)	424 (46.1)
10-14 µg/dL	201 (27.8)	17 (8.6)	218 (23.7)
15-19 µg/dL	146 (20.2)	8 (4.1)	154 (16.8)
20-44 µg/dL	113 (15.7)	4 (2.0)	117 (12.7)
45-69 µg/dL	5 (0.7)	1 (0.5)	6 (0.7)

Figures in parentheses indicate percentages. χ^2^=151.96; df=4; p<0.001; df=Degree of freedom

**Table 2.** Differences in blood lead levels by age-groups

Age-group (years)	No.	Mean±SD (µg/dL)	95% CI
<5^a^	95	12.09±9.39	10.17-14.00
5-9^b^	418	13.58±8.67	12.73-14.43
≥10^c^	406	10.84±7.26	10.13-11,55
Total	919	12.19±8.25	11.65-12.73

p value: a vs b, 0.24; a vs c, 0.37; b vs c, <0.001;

CI=Confidence interval;

SD=Standard deviation

**Table 3.** Correlation of blood lead levels with demographic variables, BMI, and haemoglobin status

Parameter		Age (year)	Sex	Income	BMI	Haemoglobin
Lead (µg/dL)	r	-0.141	0.026	0.019	-0.232	-0.102
	p	<0.001	0.436	0.587	<0.001	0.016
	n	905	905	780	848	559
Age (years)	r		0.027	-0.031	0.312	0.349
	p		0.410	0.390	<0.001	<0.001
	n		907	782	848	559
Sex (male=1, female=2)	r			-0.209	-0.095	-0.111
	p			<0.001	0.006	0.009
	n			782	848	559
Income	r				0.180	0.097
	p				<0.001	0.022
	n				726	558
BMI	r					0.219
	p					<0.001
	n					559

BMI=Body mass index

## DISCUSSION

The present study identified a high prevalence of lead poisoning, ranging from 50% to 99%, in several areas in the metropolitan city of Dhaka. Based on the recommendations of the CDC, an area is considered at a high risk if 12% or greater of children tested are found with the BLLs of ≥10 µg/dL (12). All the study areas in Dhaka, except one (Uttara), and the rural area of Chirirbandar are considered to be at a high risk of elevated lead poisoning according to the standards of the CDC. The problem of lead poisoning was significantly higher in the urban children compared to the rural children, possibly because of rapid growth of industries and their environmental contaminations in Dhaka city and the surrounding areas. Results of an earlier study showed that lead contamination in air in Dhaka city was one of the highest in the world in 1997 (13). Another study in 2003 found considerable concentrations of lead in Dhaka, despite the official ban of leaded gasoline in 1999 (14). This suggests the need for regular monitoring of industries, gasoline, and other sources of lead contaminations in the country.

**Table 4.** Risk factors for elevated blood lead levels in children in Bangladesh

Risk factor	No.	Blood lead mean±SD	p value
Industry in a close proximity to the residential area			
Present	224	13.73±5.07	<0.001
Absent	183	9.94±4.99	
Water source			
Tubewell^a^	147	13.60±5.57	a vs b=0.03
Municipal supply^b^	380	12.27±5.36	a vs c=0.001
Home filter^c^	31	9.56±4.43	b vs c=0.02
Water faucet made of			
Brass, lead, or copper	458	11.67±5.53	<0.001
Iron, plastic, or other	50	3.77±4.91	
Serving-plate made of
Melamine^d^	317	13.18±5.59	d vs e=0.001
Ceramic^e^	58	10.10±5.03	d vs f=0.05
Steel, glass, tin, other^f^	185	12.02±5.03	e vs f=0.05
Indigenous treatment			
*Kabiraji* medicine	81	13.16±6.63	0.004
Homeopathic medicine	119	10.90±4.37	

SD=Standard deviation

The risk of lead poisoning was higher in young children aged 5-9 years compared to those aged 10 years and above. Younger children are at a higher risk of lead poisoning than older ones because young children absorb lead more easily and are in a greater contact with potentially-contaminated dust and dirt (15). Another important finding was the association of high BLLs in children with low BMI, indicating that compromised nutritional status increases the risk of the problem. The direct relationship between the elevated BLLs and the poor nutritional status was also found in some other developing countries (16). A diet rich in calcium and iron is necessary because deficiencies in these minerals can increase the body's capacity for absorption of lead. This explains why malnourished children are more prone to have high levels of lead in their blood (17). However, the serum iron status or Hb values did not correlate significantly with the BLLs in this study. Another interesting finding was that children with blood group B–had significantly higher BLLs compared to those with blood group AB–. It is difficult to comment on this association, which could be trivial because of the very small sample-size of the two groups.

Other risk factors for lead poisoning identified in this study included: presence of industries in proxi-mity to the residential area; water faucets made of brass, lead, or copper; the use of melamine dinner plates; and the use of indigenous (*kabiraji*) medicines. The apparent significant correlation between the BLLs and municipal water supply on bivariate analysis was possibly due to a confounding effect of the type of residence (urban or rural setting). Once the variable—type of residence—was controlled, the BLLs and municipal water supply were no more significant. Also, results of multivariate analyses showed that the proximity to industries and the use of indigenous medicines were two significant predictors of BLLs when other confounders were controlled for. This research is unique because it is the first study reporting the possible non-occupational sources of lead contamination in Bangladesh.

One of the limitations of the study was that it did not measure the presence of lead from the sources of contamination. In addition to known sources of lead in paint, dust, and water supply, studies reported some atypical sources of lead, which included fashion accessories, folk remedies (azarcon and greta), lead-tainted ethnic cosmetics (surma), imported condiments and candies, ceramic dinnerware, lead-glazed pitchers, pellets, bullets, and mini-blinds (18). Small industries used for lead-glazed potteries and plates, flour mills, cottage industries, mining and smelting, battery repair and recycling, and car-repair workshops could be other sources of lead poisoning in developing countries (19).

### Conclusions

The challenge is a coordinated effort of the governmental and private agencies to identify specific sources and prevention of lead in the country. Due to lack of lead monitoring in Bangladeshi children, the Ministry of Health and Family Welfare should take immediate steps to routinely screen preschool and school-age children, who are at a risk of lead poisoning. Knowledge and awareness of the people about the problem of lead poisoning in developing countries, including Bangladesh, is rudimentary. The Government and the public should initiate programmes to educate people highlighting the extent of the problem of lead poisoning in the country. Based on the findings of the study, remedial measures are needed to control industrial discharges of lead in the environment.

## ACKNOWLEDGEMENTS

The study was supported by the Fulbright Scholar Grant Program 2007-2008, US Department of State, and the University of Southern Mississippi. A number of volunteers, community leaders, voluntary organizations, and private organizations contributed to data-collection: AB Foundation; Society to Uplift Social Harmony; school authorities; North South University, Dhaka; and Anwer Khan Modern Medical College Laboratory, Dhaka. The authors are thankful to Rebecca Holland for her editorial suggestions.

## References

[1] United States Environmental Protection Agency (2005). An introduction to indoor air quality (IAQ): lead (Pb).

[2] Centers for Disease Control and Prevention (2005). Preventing lead poisoning in young children.

[3] Schnaas L, Rothenberg SJ, Flores MF, Martinez S, Hernandez C, Osorio E (2006). Reduced intellectual development in children with prenatal lead exposure. Environ Health Perspect.

[4] Frisancho AR, Ryan AS (1991). Decreased stature associated with moderate blood lead concentrations in Mexican-American children. Am J Clin Nutr.

[5] Staudinger KC, Roth VS (1998). Occupational lead poisoning. Am Fam Physician.

[6] National Research Council (1993). Measuring lead exposure in infants, children, and other sensitive populations.

[7] Centers for Disease Control (1991). Preventing lead poisoning in young children.

[8] U.S. Department of Health and Human Services Healthy people 2020 summary of objectives. Environmental health: toxics and waste. EH-8: reduce blood lead levels in children.

[9] Kaiser R, Henderson AK, Daley WR, Naughton M, Khan MH, Rahman M (2001). Blood lead levels of primary school children in Dhaka, Bangladesh. Environ Health Perspect.

[10] Mitra AK, Haque A, Islam M, Bashar SAMK (2009). Lead poisoning: an alarming public health problem in Bangladesh. Int J Environ Res Public Health.

[11] Shannon M, Nader R (1997). The accuracy of a portable instrument for analysis for blood lead in children. Ambul Child Health.

[12] American Academy of Pediatrics Committee on Environmental Health (1998). Screening for elevated blood lead levels. Pediatrics.

[13] Khaliquzzaman M, Biswas SK, Tarafdar SA, Islam A, Khan AH (1997). Trace element composition of size fractionated airborne particulate matter in urban and rural areas in Bangladesh.

[14] Boman J, Gatari MJ, Wagner A, Hossain MI (2005). Elemental characterization of aerosols in urban and rural locations in Bangladesh. X-Ray Spectrom.

[15] American Academy of Pediatrics Committee on Environmental Health (2005). Lead exposure in children: prevention, detection, and management. Pediatrics.

[16] Elias SM, Hashim Z, Marjan ZM, Abdullah AS, Hashim JH (2007). Relationship between blood lead concentration and nutritional status among Malay primary school children in Kuala Lumpur, Malaysia. Asia Pac J Public Health.

[17] Bradman A, Eskenazi B, Sutton P, Athanasoulis M, Goldman LR (2001). Iron deficiency associated with higher blood lead in children living in contaminated environments. Environ Health Perspect.

[18] Gorospe EC, Gerstenberger SL (2008). Atypical sources of childhood lead poisoning in the United States: a systematic review from 1966-2006. Clin Toxicol (Phila).

[19] Falk H (2003). International environmental health for the pediatrician: case study of lead poisoning. Pediatrics.

